# Inspection of the 3’ UTR Genomic Region for RAG1 and RAG2 in Rainbow Trout (*Oncorhynchus mykiss*) Reveals Potential Regulatory Motifs

**DOI:** 10.1155/1997/81279

**Published:** 1997

**Authors:** John D. Hansen

**Affiliations:** Basel Institute for Immunology Grenzacherstrasse 487 Basel CH-4005 Switzerland

**Keywords:** RAG, enhancer, trout, regulation


					Developmental Immunology, 1997, Vol. 5, pp. 129-131
Reprints available directly from the publisher
Photocopying permitted by license only
(C) 1997 OPA (Overseas Publishers Association)
rnsterdam B.V. Published under license in The Netherlands
under the Harwood Academic Publishers imprint
part of The Gordon and Breach Publishing Group
Printed in Malaysia
Short Communication
Inspection of the 3' UTR Genomic Region for RAG1 and
RAG2 in Rainbow Trout (Oncorhynchus mykiss) Reveals
Potential Regulatory Motifs
JOHN D. HANSEN
Basel Institute fir Immunology, Grenzacherstrasse 487, CH-4005, Basel, Switzerland
(Received 27 November 1996; Infinalfirm 30 December 1996)
Keywords: RAG, enhancer, trout, regulation
In all vertebrates, antigen receptors are generated via
site-specific recombination events of germline Ig and
TCR V (D) J gene segments (Schatz et al., 1992). The
recombination activating genes and 2 (RAG1 and
RAG2) are essential for this process, which generates
the primary immune repertoire (Mombaerts et al.,
1992; Shinkai et al., 1992).' Both genes are expressed
solely in primary lymphoid tissues and precursor lym-
phocytes. The genomic locus for the RAG genes has
been conserved in both sequence and overall organiza-
tion from teleosts through humans that shared a com-
mon ancestor some 450 million years ago (Hansen and
Kaattari 1995,1996). The RAG genes are tightly linked
and are transcribed in a convergent manner utilizing a
common 3' untranslated region (5'-RAG1
--3' UTR 3'
<--- RAG2-5'). In trout, the lowest order for which the
RAG genomic locus has been characterized, the com-
mon 3' UTR is only 2.4 kb in length as compared to
5-11 kb in the higher vertebrates. In addition, the trout
RAG genes utilize overlapping polyadenylation sites,
which is unique in comparison to the higher-vertebrate
RAG loci. To date, little is known about the factors or
sequence motifs that govern the transcription of the
RAG genes. Recently, DObbeling and colleagues
(1996) showed that a genomic fragment encompassing
murine RAG1 and RAG2 contained regions responsi-
ble for their transcriptional regulation. Enhancer motifs
by definition can be located 5', 3', or within a gene it-
self, so the trout RAG 3' UTR was analyzed for se-
quence motifs that may regulate RAG transcription.
This is the first report describing the entire RAG 3'
UTR genomic sequence from any vertebrate.
Two clones encompassing the trout RAG 3' UTR
were sequenced for this study, one derived during the
isolation of RAG2 (Hansen and Kaattari, 1996) and the
other from the amplification of trout genomic DNA by
PCR using Elongase (BRL). The primers used in the
PCR (R1R2 5' CAGGAGGATGCTGACATG3' and
R2R1 5' GAAGCGCTTCTTCAGGAG 3') reactions
are located near the stop codons within the trout RAG1
and RAG2 coding sequences. The amplified products
(2.41 kb) were cloned into pCRII (Invitrogen). It was
previously reported (Hansen and Kaattari, 1996) that
the trout 3' UTR for RAG1 and RAG2 was 2.8 kb in
length, but in fact it is 2.39 kb. Both clones (Phage and
PCR-generated) were sequenced using Sequenase V1.0
(USB) and analyzed using BLAST N/X 1.4.9MP in
GCG (Altschul et al., 1990), SIGNAL SCAN V4.05
129
130 J.D. HANSEN
RAG1
AAAAAATAAATAATTTGACATTTCTTTGGAACTTTATGGATTACAGGAGGGATATGTTTTAGCAGGAGCATGTCTAATTCTAATCTGTCTGTACTGTACT
100
TTTTTTATTTATTAAACTGTAAAGAAACCTTGAAATACCTAATGTCCTCCCTATACAAAATCGTCCTCGTACAGATTAAGATTAGACAGACATGACATGA
GTTCTGCAGGGGTGGTCAGGGACCAAAAATCAGTCCTGGCATTCCTAGCATGTTAGCCCAGTGTTTTCCTTGAnGCCCACACAGCAGGCCATTTTTTCCT
101 ES/Thyl 200
CAAGACGTCCCCACCAGTCCCTGGTTTTTAGTCAGGACCGTAAGGATCGTACAATCGGGTCACAAAAGGAACTnCGGGTGTGTCGTCCGGTAAAAAAGGA
CTAGTCCCCCACACCAGCAAAACATTTTCCCTTGAGGCCCCAATTATTAGCCAGATAATGATAATTTTGTGCAAAAAAAAACAAAGTTAACATGTCCAC
201 ---+---E5---+ +---GATA1.4 300
GATCAGGGGGTGTGGTCGTTTTGTAAAAGGGAACTCCGGGGTTAATAATCGGTCTATTACTATTAAAACACGTTTTTTTTTGTTTCAATCTGTACAGGTG
TAGGCTAAAAAATGGACCCGCTAGTATAGCATTTCCACGAAAAGCCAGATGACCAGTGCGCCCCTGCTGTTCTGTTTTAATCTTAATATTTACTCTACAA
301 +---EHBo 400
ATCCGATTTTTTACCTGGGCGATCATATCGTAAAGGTGCTTTTCGGTCTACTGGTCACGCGGGGACGACAAGACAAAATTAGAATTATAAATGAGATGTT
TATGAACTATGTATTAGTAGTGATGTAAGATTAAGATACTTTATTCTGTAAATACATTATTATTAATTGTCATAATCCAAAAAAGGTTGGnTTTTTTATT
401 500
ATACTTGATACATAATCATCACTACATTCTAATTCTATGAAATAAGACATTTATGTAATAATAATTAACAGTATTAGGTTTTTTCCAACCnAAAAAATAA
TGATTCATTATTTAGAnGTGAGGACATTTGAAAATATTTTTTACCTTAAATTTAATTTCCATACATAATTCCAAAATGTTTGTTGCATTTTATATTGGTA
501 600
ACTAAGTAATAAATCTnCACTCCTGTAAACTTTTATAAAAAATGGAATTTAAATTAAAGGTATGTATTAAGGTTTTACAAACAACGTAAAATATAACCAT
TTTTATATTAGTTCATAATAATTTGATAGTGTTTTATCTCTGGGTGCCTATGAAATGTGGGACAGTTTATTGTATTGCATGGGTTATGACACATCCATGA
601 +--E2BP---+ 700
AAAATATAATCAAGTATTATTAAACTATCACAAAATAGAGACCCACGGATACTTTACACCCTGTCAAATAACATAACGTACCCAATACTGTGTAGGTACT
ATTTATCCAGTGGGTTTATTTATGGGAACAAATGAAAGCACTGTAAGTCATGGAATGGAATTGTGATAAAGGAGCTCACATTTATGATCAGTGTATTAGT
701 OCT1+ +-STAT-I--+ --GATA1.3 800
TAAATAGGTCACCCAAATAATACCCTTGTTTACTTTCGTGACATTCAGTACCTTACCTTAACACTATTTCCTCGAGTGTAAATACTAGTCACATAATCA
TTATCTCCAGTCCTGGAGCAAAATGGAATCATATAGACTCAAATTGTCTTCGTTTCAGGCTGCCATCTAAAAATATTGCTTTAGAGCTTCATATTTGGGT
801 900
AATAGAGGTCAGGACCTCGTTTTACCTTAGTATATCTGAGTTTAACAGAAGCAAAGTcCGACGGTAGATTTTTATAACGAAATCTCGAAGTATAAACCCA
CCTGAAGTcCAAAATGGGGGCCTGGGTGACTTTGAAACcATCTTTATCTGTTTTAGCATTTGCATACTGAAGGAGAAATAATACCATTcTACCTCTTTTC
901 +---GATAI.4 NFA/OCTI/2---+ 1000
GGACTTCAGGTTTTACCCCCGGACCCACTGAAACTTTGGTAGAAATAGAcAAAATCGTAAACGTATACTTCCTCTTTATTATGGTAAGATGGAGAAAAG
ATAGAATAACATATAAAGAGCCCTTTTTGTTGACCGCATGAAAATGcAGTAGGcTCCATTACTAGGcCTAACACCTCTAGGcTTGACAGAATGATGTTTT
1001 OCT1 ii00
TATCTTATTGTATATTTCTCGGGAAAAACAACTGGCGTACTTTTACGTCATCCGAGGTAATGATCCGGATTGTGGAGATcCGAAcTGTCTTACTACAAAA
TAGATGTACTTTATTGAATCCTcACACACCCTGGTTCTATGCTTCTTCTGGAGCGAGTAAACAGGGAAGcAAGTGAGCCTTGAGGGGAAcTGAAATGATT
II01 +--MZFI 1200
ATCTACATGAAATAACTTAGGAGTGTGTGGGACCAAGATACGAAGAAGACCTcGCTcATTTGTCCcTTCGTTCACTCGGAACTCCCCTTGACTTTACTAA
TGATAGTTTATTTGTTTGATTGATTGATTTTATTGTTTATCTcTGATGCCACCATTTTGAGTTGTGACAGGCAATTTTTATATTTGAcAcATTAAGTGTT
1201 +NKX2.5/GRE 1300
ACTATCAAATAAACAAACTAAcTAACTAAAATAACAAATAGAGACTAcGGTGTAAAACTCAACACTGTCCGTTAAAAATATAAAcTGTGTAATTCACAA
CTAGCTCTATTTTACTGTCATGACTTTAACCTGATGATGTACAGATTTTCATAATGTATAATATTTAGTTTAAACAACAGTCACATAATAACATCACGTT
1301 OCT1+ Myb 1400
GATcGAGATAAAATGACAGTACTGAAATTGGACTACTAcATGTCTAAAAGTATTACATATTATAAATcAAATTTGTTGTCAGTGTATTATTGTAGTGcAA
TTACATGACCGTATGTTAAATAATGTAATTTCCATTATTAAATTGcGTTTGcTTCATGAAGGATGATGGATATGGTTTGTGTGAATCTcATATcAGcGGT
1401---7 1500
AATGTACTGGCATACAATTTATTACATTAAAGGTAATAATTTAAcGCAAACGAAGTACTTCCTACTACCTATACCAAAcACACTTAGAGTATAGTCGcCA
GTATTCAATCTGTTGCTTTGTcATGTTTcAAGTGTACATTTAAACTCAGGAAAATAACAACTGTTGCCAAAAACACTCAcTCATTTGTTTTATTTGAATG
1501 NKX2.5 HOX4.3/-OCTI-+ 1600
CATAAGTTAGACAACGAAAcAGTACAAAGTTCACATGTAAATTTGAGTCCTTTTATTGTTGACAACGGTTTTTGTGAGTGAGTAAACAARTAAACTTAC
AAAATAATGATAcGTATACCTGTTGcACATTTGTTCACTGTTCACTTGTTTAATGTGTTTGTTCATTTGATTTGGTGGTTTTATTGGcCTTTATTGTTTC
1601 HOX4.3 1700
TTTTATTACTATGCATATGGACAACGTGTAAACAAGTGACAAGTGAAcAAATTACACAAACAAGTAAACTAAAcCACCAAAATAACCGGAAATAACAAAG
ACTATTAcTGAAGTATTTGTTCATCCTCTCTACAATACTGTTAATGTACTGAAAATGTAGCAAACAATTTATCACACTATTAAACTTATTCAAAATATTT
1701 1800
TGATAATGACTTCATAAACAAGTAGGAGAGATGTTATGACAATTACATGACTTTTACATCGTTTGTTAAATAGTGTGATAATTTGAATAAGTTTTATAAA
GTTGATTCTGCCATTGATAACTGAGCTTATGTGTTGGTTTATGTTATTAcACCTGTGTGAACTGATCAATGTAATTTTTTTATCATAACATATTTTGTCA
1801 +E2A like+ 1900
CAACTAAGACGGTAACTATTGACTCGAATACACAACCAAATACAATAATGTGGAcACAcTTGAcTAGTTACATTAAAAAAATAGTATTGTATAAAAcAGT
CAAACATTCAACCACAGAAAATAAATTATATTCTATAGGTTTACATACAGTTGTGCCAGTCCAAGCAGTATTAGCCTAcCTTCCAAAGAAAATTTGGCTT
1901 2000
GTTTGTAAGTTGGTGTCTTTTATTTAATATAAGATATCCAAATGTATGTCAACACGGTCAGGTTCGTCATAATCGGATGGAAGGTTTCTTTTAAACCGAA
GAcAAACCTCAAATATTCTAAAAGAATAATGAAGAATAATGACTGAcAAAAACGAGGGTGAAcAAAGCAAGCCAcAAAGTcACTATGTGTAATATGTGGG
2001- .B enh 2100
CTGTTTGGAGTTTATAAGATTTTCTTATTAcTTCTTATTACTGAcTGTTTTTGcTcCCACTTGTTTcGTTCGGTGTTTCAGTGATACAcATTATACACCC
AGTAGAACATGAAGCAAATAGACTCTGGGATTAATTTCCATTTTTATTCAATTCGGTTTCATACATTTTTTGGCAAAACTTTAGTTTATTGCATAAAATA
2101 3b-+ Ikaros 2200
TCATCTTGTACTTCGTTTATCTGAGACCCTAATTAAAGGTAAAAATAAGTTAAGCCAAAGTATGTAAAAAACCGTTTTGAAATCAAATAACGTATTTTAT
TTGAACACTATAAGGAATCAAAAAACAAGGAACAAAAAATTATTCAGTTTACTTATAAGTTTGAGAACAGTAAATTGCTGCAATGGCACATCAAACAATG
2201 5-+ 2300
AACTTGTGATATTCCTTAGTTTTTTGTTCCTTGTTTTTTAATAAGTCAAATGAATATTCAAACTCTTGTCATTTAACGACGTTACCGTGTAGTTTGTTAC
ATATGGCATTTAGGCTATAATTAGAATGACATATTTTGAACCAGTTGATCAATGAGTAAGTATAACTATACTGCATGGTCAGACATT-3'
2301 $8 homeodomain 2389
TATACCGTAAATCCGGTATATTAATCTTACTGTAT%ACTTGGTCAATAGTTACTATTCATATTGATATGACGTACAGTCTGTAA-5 RAG
FIGURE Sequence of the common 3' untranslated region for the trout RAG locus depicting potential motifs (underlined on appropriate
strand) involved in transcriptional regulation. The strand and orientation for RAG1 and RAG2 are depicted in bold with arrows.
Polyadenylation sites are in bold (Hansen and Kaattari, 1996).
RAG 3' UTR REGION IN TROUT 131
(Prestridge, 1991), and MatInspector V2.0/TRANS-
FAC 3.0 matrix (Quandt et al., 1995), both of which
can be found on the WWW. As a control for motif-
recognition specificity, the genomic sequence for trout
RAG1 (3.9 kb) was used, which detected three con-
served motifs, AP fos/jun, 3' NFKB enhancer, and the
T-cell-specific Lyf- motif.
Both 3' UTR clones were identical in size (2,389 bp)
and only displayed 4-bp differences attributable to PCR
errors. BLASTN/X searches did not reveal any major
significant homologies. An inspection of the 3' UTR
(Fig. 1) using Signal Scan and MatInspector
(matrix/core 0.97) motif-recognition programs
found an array of regulatory motifs (GATA, NFA,
E2BP, Oct-l/2, NF-KB-en, AP1, STAT-1, Ikaros-2,
NKX-2.5 (Tinman homologue), glucocorticoid re-
sponse elements (GRE), HOX and cutlike home-
odomain binding sites involved in lymphocyte regula-
tion, development, and viral replication. Some of these
elements (gE5, laB, and Octamers) were also found in
the catfish IgH locus (Magor et al., 1994), which were
shown to regulate tissue-specific expression in murine-
transfected lymphocytes. Additionally, two variants of
the MHC DRA X box were found, which are primarily
utilized by MHC class II+ cells. Finally, it is indeed
intriguing to note that both B- and T-cell-specific ele-
ments (Octamers and GATA1.3) were found within the
trout RAG intergenic regior, which may be related in
some way to the lymphoid-specific expression of the
RAG genes.
Whether the motifs described in this paper are essen-
tial or conserved in all vertebrates will have to await
further analysis to determine their potential role in V
(D) J recombination. Future efforts will include re-
porter constructs containing the trout RAG 3' UTR to
discern if it can regulate transcription in a tissue/lym-
phoid-specific manner.
Acknowledgements
I would like to thank Drs. Jos6 A. Garcia-Sanz and
Kerry Campbell for their comments on this manu-
script. The Basel Institute for Immunology was found-
ed and is supported by Hoffman-LaRoche Ltd., Basel,
CH.
The nucleotide sequence data reported in this paper
have been submitted to the GenBank nucleotide se-
quence database and have been assigned the accession
number U73750.
References
Altschul, S. E, Gish, W., Miller, W., Myers, E. W., and Lipman, D.
J. (1990). Basic local alignment tool. J. Mol. Biol., 215,403-410.
D/Sbbeling, U., Hobi, R., Berchtold, M. W., and Kuenzle, C. C.
(1996). V (D) recombination is regulated similarly in RAG-
transfected fibroblasts and pre-B cells. J. Mol. Biol., 261,
309-314.
Hansen, J. D., and Kaattari, S. L. (1995). The recombination acti-
vating.gene (RAG1) of rainbow trout (Oncorhynchus mykiss):
Cloning, expression and phylogenetic analysis. Immunogenetics,
42, 188-195.
Hansen, J. D., and Kaattari, S. L. (1996). The recombination acti-
vating gene 2 (RAG2) of the rainbow trout Oncorhynchus
mykiss. Immunogenetics, 44,203-211.
Magor, B. G., Wilson, M. R., Miller, N. W., Clem, L. W., Middle-
ton, D. L., and Warr, G. W. (1994). An Ig heavy chain enchancer
of the channel catfish Ictalurus punctatus: Evolutionary conser-
vation of function but not structure. J. Immunol., 153,
5556-5563.
Mombaerts, P., Iacomini, J., Johnson, R. S., Herrup, K., Tonegawa,
S., and Papaioannou, V. E. (1992). RAG-1 deficient mice have no
mature B and T lymphocytes. Cell, 68, 869-877.
Prestridge, D. S. 1991 ). SIGNAL SCAN: A computer program that
scans DNA sequences for eukaryotic transcriptional elements.
CABIOS, 7, 203-206.
Quandt, K., Frech, K., Karas, H., Wingender, E., and Werner, T.
(1995). MatInd and MatInspector: New fast and versatile tools
for detection of consensus matches in nucleotide sequence data.
Nucleic Acids Res., 23, 4878-4884.
Schatz, D. G., Oettinger, M. A., and Schlissel, M. S. (1992). V (D)
recombination: Molecular biology and regulation. Annu. Rev.
lmmunol., 10,359-383.
Shinkai, Y., Rathbun, G., Kong-Peng, Y., Oltz, E. M., Stewart, V.,
Mendelsohn, M., Charron, J., Datta, M., Young, F., Stall, A. M.,
and Alt, E W. (1992). RAG2-deficient mice lack mature lympho-
cytes owing to inability to initiate V (D) rearrangement. Cell,
68, 855-867.

				

